# Early Diagnosis of COVID-19 on Non-Chest CT Studies in the Emergency Setting: A Case Series and Review of the Literature

**DOI:** 10.7759/cureus.11748

**Published:** 2020-11-28

**Authors:** Rishabh Gattu, Tejasvi Kainth, Gagandeep Singh, Nicole M Sakla, Michael Sadler

**Affiliations:** 1 Radiology, Newark Beth Israel Medical Center, Newark, USA; 2 Language Access and Internal Medicine, Winnipeg Regional Health Authority, Winnipeg, CAN

**Keywords:** covid-19, corona virus, non-ct chest, ground-glass opacities

## Abstract

Severe acute respiratory syndrome coronavirus 2 (SARS-CoV-2) is a novel strain of coronavirus that has spread throughout the globe causing coronavirus disease 2019 (COVID-19). As the number of cases rises in the United States (US), it has become more imperative to detect COVID-19 at its earliest radiologic stage to decrease community transmission.

In this case series, we discuss five patients who presented with non-respiratory-related symptoms and underwent non-chest CT imaging, such as abdominal and neck CT, with a portion of the lungs visualized in each respective study.

Imaging findings of COVID-19 include basilar and peripherally predominant pulmonary parenchymal ground-glass opacities. All five of our patients had findings suggestive of COVID-19 that prompted the radiologist to suggest testing for the disease. Subsequently, four of the five patients tested positive for COVID-19, and one of them was presumed to have the diagnosis based on clinical and imaging findings.

## Introduction

On January 7, 2020, severe acute respiratory syndrome coronavirus 2 (SARS-CoV-2) was isolated from a human for the first time, associated with the exposure within the seafood market in Wuhan, China [1]. As of June 21, 2020, the total number of confirmed coronavirus disease 2019 (COVID-19) cases in the United States (US) stands at 2,208,829, and there have been 118,895 COVID-19-related deaths [2]. COVID-19 pneumonia manifests in variable ways, showing peripheral parenchymal ground-glass opacities, pleural effusions, and pulmonary nodules. Less commonly, the findings include bronchial wall thickening, interlobular and interlobular septal thickening, linear opacities, and "reverse halo" sign [3,4].

We present a series of five patients who underwent imaging for non-respiratory-related symptoms, where a portion of the lung was in the field of imaging. These patients had findings suggestive of COVID-19, which prompted the radiologist to advise the clinical team to conduct testing for COVID-19. Ultimately, nearly every patient was found positive for SARS-CoV-2 via reverse transcription-polymerase chain reaction (RT-PCR).

## Case presentation

Case 1

**Figure 1:**
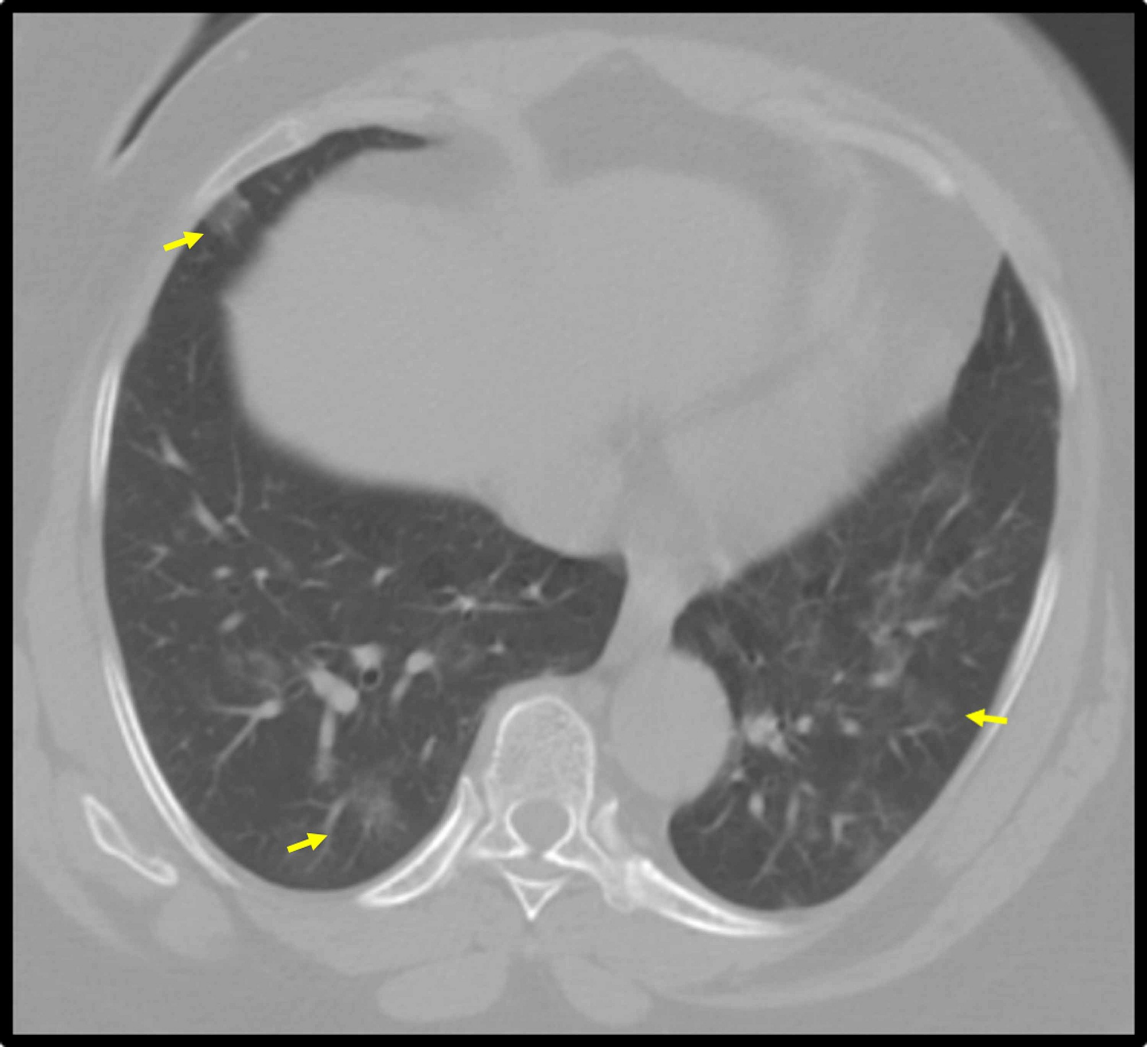
Axial image from a CT of the abdomen and pelvis without contrast through the lung bases The image shows peripherally predominant ground-glass opacities bilaterally (yellow arrows) CT: computed tomography

A 65-year-old female with a history of hypertension and obesity presented to the emergency department (ED) complaining of dizziness and left-flank pain. The patient reported mild dyspnea on exertion, as well as the loss of smell and taste, but denied fever, nausea, vomiting, or chest pain. She reported that her roommate had died of COVID-19 infection two days prior to the admission. No opacities were found on the chest radiograph. A CT of the abdomen and pelvis without contrast was performed, and no acute abdominal findings were identified; however, small peripheral ground-glass opacities appeared throughout the visualized lung bases (Figure 1). The patient was admitted to the COVID unit, and her COVID-19 RT-PCR viral nasal swab returned positive. The patient was subsequently treated with hydroxychloroquine and IV methylprednisolone. She clinically improved with minimal oxygen support via nasal cannula.

Case 2

A 73-year-old male with a history of end-stage renal disease, hypertension, diabetes, and congestive heart failure presented to the ED for mild dyspnea. An initial chest radiograph was negative for pneumonia, and he was discharged home.

**Figure 2:**
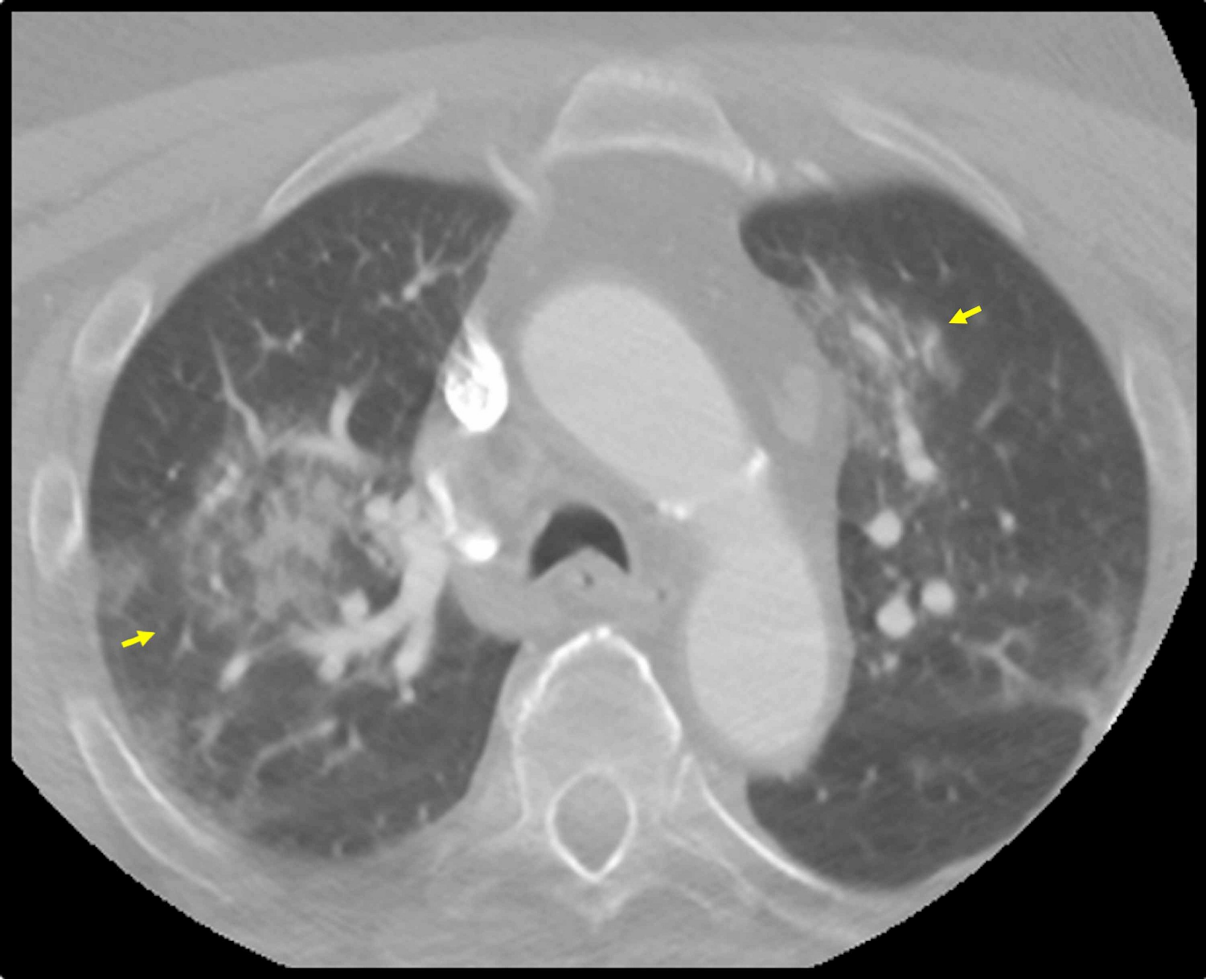
Axial image from a CT angiography of the head and neck at the level of the aortic arch The image displays randomly distributed ground-glass opacities bilaterally (yellow arrows) CT: computed tomography

Three days later, he returned to the ED with right-sided weakness and facial droop. Unable to provide further history, he was sent for an immediate CT head and CT angiography of the head and neck for stroke evaluation. The CT head revealed a chronic right cerebellar infarct without hemorrhage or evidence of acute infarct. On CT angiography, the lung apices contained patchy ground-glass opacities involving the nondependent upper lobes bilaterally, which raised suspicion for COVD-19 (Figure 2). MRI brain was not performed.

His treatment consisted of urgent dialysis and aspirin for metabolic encephalopathy and acute stroke, respectively. COVID-19 RT-PCR viral nasal swab was performed to confirm CT findings and the test returned positive. The patient was treated with doxycycline for COVID-19 pneumonia. Within the next few days, his respiratory status declined, requiring increasing amounts of supplemental oxygen. The patient ultimately died from acute hypoxic respiratory failure secondary to COVID-19 pneumonia.

Case 3

**Figure 3:**
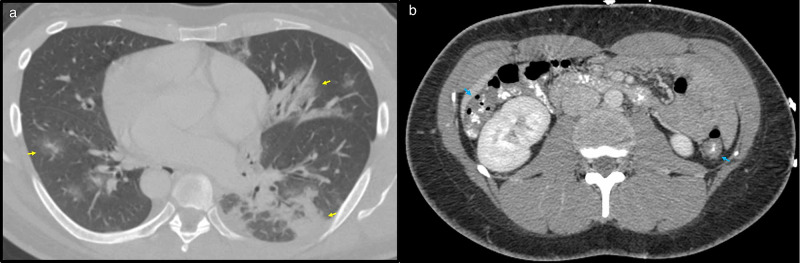
Axial image from a contrast-enhanced CT of the abdomen and pelvis (a) The image shows ground-glass opacities (yellow arrows) bilaterally at the lung bases and patchy consolidations (red arrows) in the left lower lobe. (b) In the upper abdomen, there is mild circumferential wall thickening of the transverse and descending colon (blue arrows) CT: computed tomography

A 30-year-old female presented to the ED with complaints of nausea and diffuse abdominal pain. She had no known COVID-19 exposure or history of recent travel. She denied respiratory symptoms and did not require supplemental oxygen. A contrast-enhanced CT of the abdomen and pelvis revealed mild diffuse colonic wall thickening to suggest inflammatory or infectious colitis and partially imaged ground-glass opacities at the lung bases (Figure 3). The patient was not tested at the hospital for COVID-19 given the absence of clinical concern. Instead, based on the radiologist’s suggestion of COVID-19/viral pneumonia, the patient was counseled in the ED about a 14-day self-quarantine, social distancing, proper hygiene, and instructions for outpatient COVID-19 testing. After discharge, she returned three days later for similar symptoms.

Case 4

**Figure 4:**
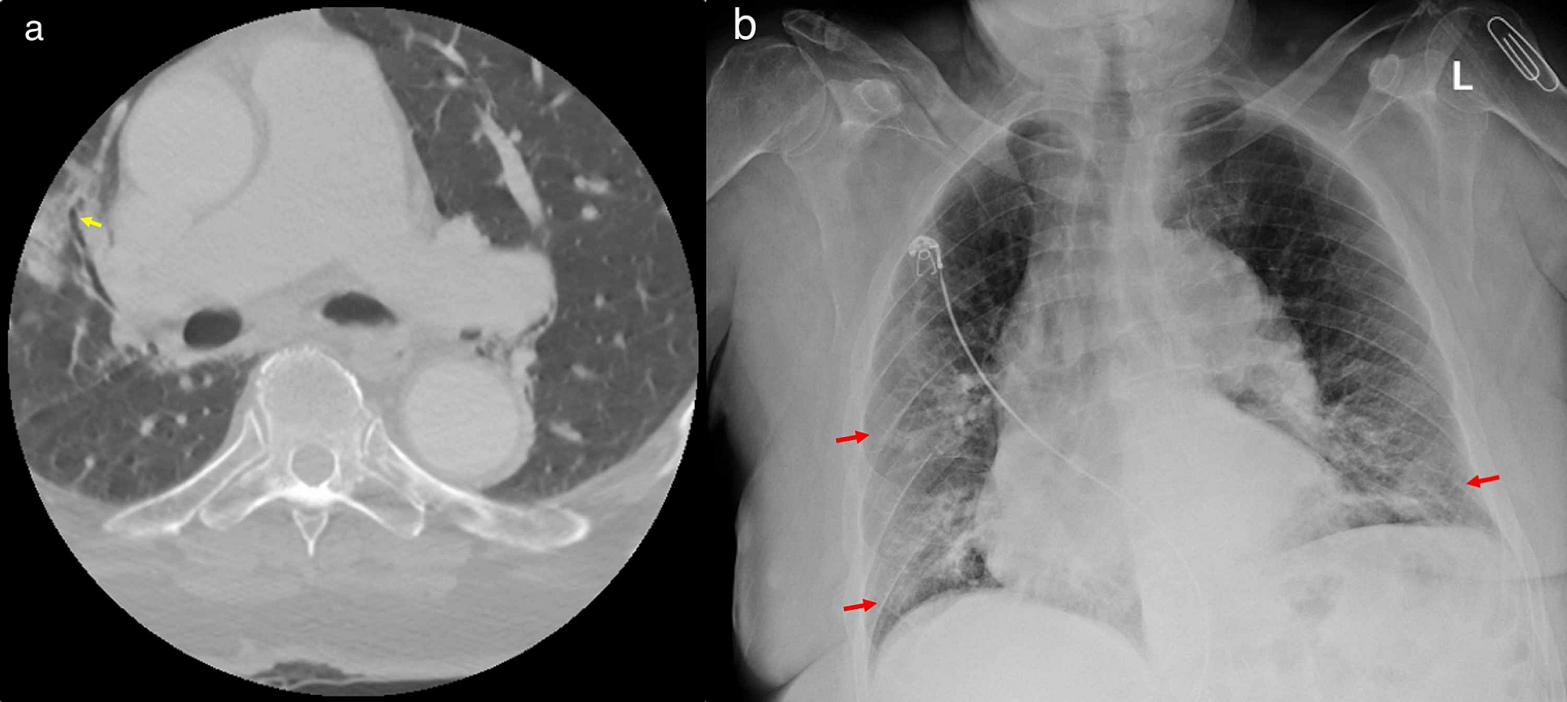
CT and chest radiograph of the patient (a) Axial image from a CT cervical, thoracic, and lumbar spine without contrast at the level of the mid lungs displays ground-glass infiltrates in the right middle lobe (yellow arrows). (b) Subsequent chest radiograph demonstrates bilateral lung infiltrates, asymmetric to the right (red arrows) CT: computed tomography

A 77-year-old male with no known comorbidities presented to the ED with complaints of bilateral leg weakness, back pain due to multiple falls, and malaise. Due to concern for trauma, non-enhanced CT of the cervical, thoracic, and lumbar spine was performed, revealing no fractures or malalignment. Limited views of the lungs showed patchy ground-glass infiltrates in the right lung, suggestive of COVID-19 pneumonia (Figure 4a). Further assessment with a chest radiograph demonstrated diffuse, right-greater-than-left, lung infiltrates (Figure 4b), and COVID-19 RT-PCR viral nasal swab was positive.

On presentation, the patient was mildly hypoxic, requiring supplemental oxygen via nasal cannula. Despite oxygen support and a five-day hydroxychloroquine course, the patient died two weeks later from hypoxic respiratory failure secondary to COVID-19 pneumonia.

Case 5

**Figure 5:**
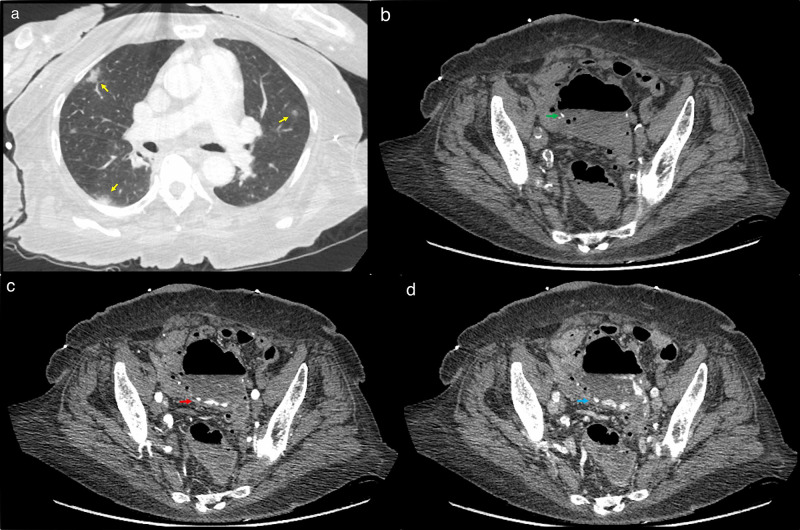
Radiological findings in patient 5 Axial image from CT angiogram of the abdomen and pelvis (a) at the lung bases shows peripheral ground-glass opacities bilaterally (yellow arrows). Axial image in (b) non-contrast phase shows an ileosigmoid anastomosis (green arrow), with (c) intravenous contrast extravasation in the arterial phase (red arrow) and (d) pooling (blue arrow) in the sigmoid bowel lumen distal to the anastomosis on venous phase to suggest active gastrointestinal bleeding. The source appears to arise from an inferior mesenteric artery branch CT: computed tomography

An 85-year-old female with multiple comorbidities, including end-stage renal disease, hypertension, peripheral arterial disease, prior gastrointestinal bleed requiring subtotal colectomy, and endometrial cancer, was referred from her outpatient dialysis clinic for massive rectal bleeding with passage of clots. CT angiography of the abdomen and pelvis was completed for evaluation of gastrointestinal bleed and revealed active bleeding into the sigmoid colon (Figure 5).

There was an incidental finding of peripherally oriented ground-glass opacities at the lung bases, which was suggestive of COVID-19 pneumonia according to the interpreting radiologist (Figure 5). The patient later tested positive for COVID-19 via an RT-PCR viral nasal swab. Subsequently, a five-day course of hydroxychloroquine was administered.

Her hospital course was complicated by hemorrhagic shock, as she required seven units of packed red blood cells, three units of fresh frozen plasma, and three bags of platelets. She was taken to the interventional radiology suite, with the staff enacting droplet precautions, due to lung findings on CT, for angiography and embolization. The mesenteric angiogram showed an active bleed in the sigmoid branch of the inferior mesenteric artery, for which particulate embolization was performed until hemostasis was achieved.

Over the course of one week, the patient’s respiratory and hemodynamic status declined. Given her multiple comorbidities indicative of poor prognosis, a decision was made to transition the patient to comfort care.

## Discussion

It has been several months since COVID-19 was declared a global pandemic, and there is a potential for a second wave of increasing SARS-CoV-2 cases [5]. The US is witnessing an increase in the number of new cases as the lockdown measures are lifted.

Studies have found that RT-PCR assay is 71% sensitive to detect COVID-19 during the early disease process, while chest CT has a sensitivity of 98% in detecting COVID-19 during the early disease process [6]. The Centers for Disease Control and Prevention (CDC) recommend collecting and testing specimens from the respiratory tract as part of initial diagnostic testing. It is suggested that findings on chest imaging in COVID-19 are not specific and overlap with other viral infections. However, the American College of Radiology (ACR) advises radiologists to familiarize themselves with CT findings of COVID-19 infection in order to identify findings consistent with infection in patients imaged for other reasons [7]. This allows the clinical team to isolate the patient and prevent the spread of infection, which can help reduce the infection rate during the anticipated second wave. 

Non-chest CT examinations established the diagnosis of COVID-19-related pneumonia in 76 out of 119 (64%) patients in a retrospective study that consisted of 101 CT abdomen/pelvis and 18 CT cervical spine/neck [8]. The most common finding was the presence of peripheral ground-glass opacities in 96% of patients, followed by consolidation in 40% and interlobular and interlobular septal thickening in 23% of patients. Air-trapping was observed in 5%, bronchiectasis in 6.7%, pleural effusions were reported in 11%, and pleural thickening was noted in 6.7% of the patients. This study reported that patients who had COVID-19 detected on CT cervical spine/neck had statistically significant worse outcomes compared to those who underwent CT abdomen/pelvis, possibly due to the infection's neurologic sequelae, such as acute cerebrovascular injury [8].

Dane et al. identified unanticipated lung base findings from CT abdomen/pelvis examinations suggestive of COVID-19 [9]. Ground-glass opacity was the most common lung base finding observed. The most common location of ground-glass opacities was multi-lobar followed by peripheral and peribronchovascular, while other findings included consolidation and ground-glass nodules [9].

In this case series, the majority of patients presented with non-respiratory symptoms, for which imaging was performed to evaluate non-chest-related pathology. In each case, positive findings in partially imaged lung parenchyma suggested the diagnosis of COVID-19 pneumonia, which was confirmed by COVID-19 nasal swab. One exception was the 30-year-old female patient who presented with abdominal pain: the diagnosis was made by imaging features at a time when no set standard of care existed regarding the necessity of diagnostic testing. The diagnosis was deemed clear in the setting of high incidence and reported cases of extra-pulmonary manifestations of COVID-19 [10,11]. Furthermore, this same patient’s CT contained an additional patchy consolidation in the left lower lobe, a feature that did not portend a poorer prognosis. Nonetheless, all five patients diagnosed with COVID-19 pneumonia in our case series possessed ground-glass infiltrates in some distribution and degree, which is consistent with the trend described in the aforementioned literature [8,9].

Imaging findings for the 73-year-old patient who presented with stroke-like symptoms held prognostic value. The initial chest radiography three days prior to his second ED visit was negative, a testament to the poor sensitivity of radiography in detecting early COVID-19 pneumonia. Additionally, Yu et al. have concluded that upper lung zone involvement (right upper lobe, right middle lobe, left upper lobe) correlated with increased mortality rates and that an increased pulmonary lesion in the bilateral upper lungs was an independent risk factor for adverse clinical outcomes [12]. This patient's consolidation superimposed on ground-glass opacities may have represented the progression of the disease, and upper lung zone involvement deemed him a high risk for adverse outcomes. Multiple comorbidities and upper lung involvement contributed to this patient’s eventual demise. Thus, early diagnosis may have proven to be crucial in determining a proactive management approach.

## Conclusions

We identified a subset of patients who showed no initial respiratory symptoms as their chief complaint but demonstrated subtle characteristic lung parenchymal changes on non-chest CT studies. These imaging findings can be used to diagnose asymptomatic patients carrying SARS-CoV-2 in the early stages. Ultimately, these patients can be quarantined earlier to attempt to decrease community transmission of the virus and potentially mitigate the impact of a “second wave.”

## References

[1] (2020). World Health Organization: novel coronavirus (2019-nCoV) situation report. https://www.who.int/docs/default-source/coronaviruse/situation-reports/20200121-sitrep-1-2019-ncov.pdf.

[2] (2020). World Health Organization: coronavirus disease 2019 (COVID-19) situation report - 153. https://www.who.int/docs/default-source/coronaviruse/situation-reports/20200621-covid-19-sitrep-153.pdf?sfvrsn=c896464d_2.

[3] Chung M, Bernheim A, Mei X (2020). CT imaging features of 2019 novel coronavirus (2019-nCoV). Radiology.

[4] Vu D, Ruggiero M, Choi WS, Masri D, Flyer M, Shyknevsky I, Stein EG (2020). Three unsuspected CT diagnoses of COVID-19. Emerg Radiol.

[5] Wise J (2020). Covid-19: risk of second wave is very real, say researchers. BMJ.

[6] Fang Y, Zhang H, Xie J, Lin M, Ying L, Pang P, Ji W (2020). Sensitivity of chest CT for COVID-19: comparison to RT-PCR. Radiology.

[7] Radiology ACo (2020 (2020). ACR recommendations for the use of chest radiography and computed tomography (CT) for suspected COVID-19 infection. https://www.acr.org/Advocacy-and-Economics/ACR-Position-Statements/Recommendations-for-Chest-Radiography-and-CT-for-Suspected-COVID19-Infection.

[8] Hossain R, Lazarus MS, Roudenko A (2020). CT scans obtained for nonpulmonary indications: associated respiratory findings of COVID-19. Radiology.

[9] Dane B, Brusca-Augello G, Kim D, Katz DS (2020). Unexpected findings of coronavirus disease (COVID-19) at the lung bases on abdominopelvic CT. AJR Am J Roentgenol.

[10] Chaudhry F, Kainth T, Sakla NM, Singh G, Marian V (2020). Extrapulmonary gastrointestinal presentation of coronavirus (COVID-19): a case report and review of literature. Cureus.

[11] Pan L, Mu M, Yang P (2020). Clinical characteristics of COVID-19 patients with digestive symptoms in Hubei, China: a descriptive, cross-sectional, multicenter study. Am J Gastroenterol.

[12] Yu Q, Wang Y, Huang S (2020). Multicenter cohort study demonstrates more consolidation in upper lungs on initial CT increases the risk of adverse clinical outcome in COVID-19 patients. Theranostics.

